# Social Problem-Solving and Suicide Risk Across the Lifespan: A Qualitative Investigation

**DOI:** 10.1093/geroni/igaa057.1029

**Published:** 2020-12-16

**Authors:** Ali Molaie, Adrienne Chong, Jane Fisher

**Affiliations:** University of Nevada, Reno, Reno, Nevada, United States

## Abstract

Social disconnectedness, or thwarted belongingness (TB) is a risk factor for suicide across the lifespan. Perceived time left (PTL) to live may impact perceptions of hopefulness for future social connectedness, and thus the generation of problem-solving strategies for decreasing TB and the risk for suicide. We conducted an exploratory analysis of the quantity and quality of older and younger adults’ recommendations for decreasing TB of characters in vignettes who were depicted as having high or low TB (i.e., described as lonely or not lonely) and high or low PTL (i.e., described as being 35-years-old or 85-years-old). We also examined the relation between participants’ social recommendations and suicide risk. Participants with higher suicide risk endorsed higher hopelessness on the High TB/Low PTL vignette and were more likely to recommend contact with family as a means of reducing TB (χ^2(1) = 4.25, p = 0.04) than were participants with lower suicide risk. Participants with higher suicide risk were also more likely to recommend social activity with friends than were participants with lower suicide risk on the High TB/High PTL vignette, (χ^2(1) = 6.66, p = 0.01) and on the High TB/Low PTL vignette (χ^2(1) = 6.14, p = 0.02). Additionally, age differences were observed such that older participants made fewer recommendations involving socializing with family and friends than did younger adults, and provided more nonsocial or made no recommendations across vignettes. The results suggest that social problem-solving may be an important differentiating variable among older and younger adults at risk for suicide.

